# Detection of Modified Forms of Cytosine Using Sensitive Immunohistochemistry

**DOI:** 10.3791/54416

**Published:** 2016-08-16

**Authors:** Abdulkadir Abakir, Lee Wheldon, Andrew D. Johnson, Patrick Laurent, Alexey Ruzov

**Affiliations:** ^1^Laboratoire de Neurophysiologie (CP601), ULB Neuroscience Institute (UNI), Université Libre de Bruxelles; ^2^Medical Molecular Sciences, Centre for Biomolecular Sciences, University of Nottingham; ^3^School of Life Sciences, University of Nottingham; ^4^Division of Cancer and Stem Cells, Centre for Biomolecular Sciences, School of Medicine, University of Nottingham

**Keywords:** Molecular Biology, Issue 114, Brain tissues, DNA methylation (5mC), DNA demethylation, 5-hydroxymethylcytosine (5hmC), 5-formylcytosine (5fC), 5-carboxylcytosine (5caC), Immunohistochemistry, Signal amplification, Immunofluorescence

## Abstract

Methylation of cytosine bases (5-methylcytosine, 5mC) occurring in vertebrate genomes is usually associated with transcriptional silencing. 5-hydroxylmethylcytosine (5hmC), 5-formylcytosine (5fC), and 5-carboxylcytosine (5caC) are the recently discovered modified cytosine bases produced by enzymatic oxidation of 5mC, whose biological functions remain relatively obscure. A number of approaches ranging from biochemical to antibody based techniques have been employed to study the genomic distribution and global content of these modifications in various biological systems. Although some of these approaches can be useful for quantitative assessment of these modified forms of 5mC, most of these methods do not provide any spatial information regarding the distribution of these DNA modifications in different cell types, required for correct understanding of their functional roles. Here we present a highly sensitive method for immunochemical detection of the modified forms of cytosine. This method permits co-detection of these epigenetic marks with protein lineage markers and can be employed to study their nuclear localization, thus, contributing to deciphering their potential biological roles in different experimental contexts.

**Figure:**
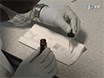


## Introduction

Methylation of cytosine bases in DNA (5mC) represents a major epigenetic mark found in vertebrates genomes associated with transcriptional silencing^1^. 5mC is being introduced and maintained by DNA methyltransferases^2-5^, and has been shown to play important roles in a number of biological processes including genomic imprinting, X-chromosome inactivation, cellular differentiation, and development^3, 6^. Consequently, the disruption of 5mC genomic patterns is associated with a number of diseases^7, 8-11^. Despite the progress in understanding 5mC role in development and disease, it is still remain largely unknown how this mark is being removed in developing and adult tissues. Several potential mechanisms of DNA demethylation have been recently proposed including active and passive demethylation mechanisms^12, 13, 14, 15^. Discovery of the products of 5mC sequential oxidation mediated by ten-eleven translocation enzymes (tet1/2/3) such as 5-hydroxylmethylcytosine (5hmC), 5-formylcytosine (5fC), and 5-carboxylcytosine (5caC) in eukaryotic DNA ^16, 17, 18, 19 ^prompted speculations whether they may serve as intermediates of DNA demethylation processes or act as stable epigenetic marks in their own right^13^. While the demonstration that the component of base excision repair, thymine-DNA glycosylase (TDG) can bind and remove both 5fC and 5caC from DNA^19, 20^ suggests a role for the modified 5mC derivatives in an active DNA demethylation. Recent evidence showing that 5fC/5caC can modulate the rate of RNA II processivity points to potential involvement of these marks in transcriptional regulation^29^. Due to this potential biological importance of oxidized forms of 5mC, a range of biochemical and antibody based techniques have been employed for studying their genomic distribution and global content^16, 19-24^.

Given that most of the vertebrate organs consist of different cell types and that the distribution of modified cytosine bases is tissue and cell-type specific ^16-18, 20, 23, 25-27^, determining the spatial distribution of oxidized 5mC derivatives in different tissues becomes an important experimental task necessary for unveiling their biological functions. Most of the biochemical and antibody based approaches do not provide any spatial information regarding the distribution of the modified forms of 5mC in different tissue and cell-types. In contrast, immunochemistry based techniques can provide a rapid tool for assessing the spatial distribution and nuclear localization of 5mC^28^ and its oxidized derivatives^20^. That is said, the reported very low abundance of 5fC (20 in every 10^6^ cytosine) and 5caC (3 in every 10^6 ^cytosine) in the mouse genome^18 ^represents a significant challenge for standard immunochemistry.

Here, we describe a highly sensitive immunochemical method that provides robust and a rapid detection of oxidized form of cytosine in mammalian brain tissue. By incorporating peroxidase-conjugated secondary antibodies coupled with a signal amplification step, this method circumvents the challenges of detecting the very low amounts of 5fC and 5caC. In addition, this technique can be used to co-detect the modified forms of cytosine with lineage specific markers, effectively complementing other approaches in elucidating the biological functions of these epigenetic marks.

## Protocol

All the animal-involved procedures were performed in accordance with the University of Nottingham's ethical review board.

### 1. Selecting Suitable Tissue Preparation for Immunostaining

1. Generate paraffin-embedded section of wild-type CD1 mouse embryos and adult brain tissues as described previously^25^. Use brain tissue sections fixed with either 4% formaldehyde (FA) or 4% paraformaldehyde (PFA) for immunostaining method^25^. **NOTE:** The use of paraffin embedded tissue sections requires de-waxing prior to antibody labelling. Since the treatment with 2 - 4 M hydrochloric acid (HCl) employed in the protocol for DNA denaturing is not compatible with most antigen retrieval strategies, we recommend the use of either cryo- or microtome sections for co-detection of 5mC oxidation derivatives with protein markers.

### 2. Dewaxing Paraffin Embedded Tissue Sections

1. In a running class II safety cabinet, wash the paraffin embedded tissue sections in a Coplin jar filled with xylene, 2 times for 10 min each at RT. **NOTE:** It is important to use fresh xylene as incomplete paraffin removal can lead to inconsistent staining patterns.
2. After de-waxing, rapidly rehydrate the tissue sections by washing consecutively in 95, 75, and 50% ethanol for 10 min each at RT.

### 3. Fixation and Permeabilization of Cryo- and Microtome Sections

1. Fix the rehydrated tissue sections by placing them in either ice cold 4% PFA or 4% FA for 15 min at RT.
2. Remove excess fixative by washing the sections in PBS (Phosphate buffer saline) for 5 min at RT.
3. Permeabilize the tissue sections by placing in a Coplin jar filled with PBX (0.5% Triton X-100 in PBS) for 30 min at RT. Remove excess PBX by washing the sections shortly in PBT (0.01% Tween 20 in PBS).

### 4. Immunostaining for Oxi-5mC Derivatives

1. Place the permeabilized-sections in 2 N HCl for 60 min at RT for depurination of the DNA. **NOTE:** Although higher concentrations of HCl (*e.g.,* 4 N) lead to a more efficient DNA denaturing, they are not compatible for co-detection of oxi-mCs with DAPI.
2. Place the sections in 10 mM Tris-HCl (pH 8.5) for 30 min at RT to neutralize the HCl. Alternatively, wash the sections three times for 5 mins each in PBS. Incubate the sections in PBT for 5 min at RT.
3. Carefully remove the liquid from the area surrounding the tissue section without letting the tissue section to dry completely at any step by gently shaking the slide. Use a hydrophobic barrier pen to encircle the section without touching the section. **NOTE:** The hydrophobic barrier pen reduces the volume of antibody required to stain the tissue and can allow multiple sections to be stained with different antibodies on the same slide.
4. Incubate sections in 100 µl of blocking solution (10% bovine serum albumin in PBS) for 1 hr at RT in a humid chamber. **NOTE:** Skipping this step would not affect the efficiency of staining the modified cytosine bases.
5. Incubate the tissue sections in 100 µl of a 1:5,000 dilution of mouse monoclonal anti-5hmC and a 1:1,000 dilution of rabbit polyclonal anti-5caC primary antibodies in blocking solution for 1 hr at RT in a humid chamber. Alternatively, perform the incubation overnight at 4 °C if needed. **NOTE:** Sections processed without primary antibodies can serve as suitable negative controls for the staining procedure. The 12.5 days post coitum murine embryonic brain sections enriched in 5caC, 5fC and 5hmC^ 20^ can be used as positive control.
6. Remove excess antibodies by washing the sections in a Coplin jar filled with PBT three times for 5 min each in a Coplin jar at RT. **NOTE:** Increasing the volume of washing solutions can reduce background staining.
7. Remove excess PBT and, if necessary, encircle the sections again with the hydrophobic barrier pen as PBT contain detergent that can weaken the hydrophobic barrier.
8. Make a 1:400 dilution of goat anti-rabbit HRP-conjugated antibody and a 1:400 dilution of donkey anti-mouse 555-conjugated antibody in blocking solution.
9. Incubate the tissue sections in 100 µl of secondary antibody mixture from **step 4.9** for 1 hr at RT in a humid chamber.
10. Wash the tissue sections in a Coplin jar filled with PBT three times for 5 min each at RT.
11. Place the tissue sections in 100 µl of a 1:200 dilution of tyramide in the tyramide signal amplification buffer for 2 min at RT.
12. **Immediately,** remove excess tyramide solution by washing the slides three times for 5 min each in PBT.
13. Carefully remove excess PBT and **immediately** cover the sections with a drop of a Mounting Medium (see Materials List).
14. Gently place a coverslip on the tissue sections and **immediately** seal the coverslip with nail polish.
15. Store the tissue sections at 4 °C for several hr before microscopic examination. Examine under a fluorescent microscope at 405, 488 and/or 555 nm (10 - 40X magnification).

## Representative Results

To determine the distribution of 5hmC in brain tissue sections, we performed co-detection of this epigenetic modification with a marker for post-mitotic neurons, NeuN, employing commercial anti-5hmC antibody that specifically interacts with this mark but not with other forms of modified cytosine^20, 25^. Immunohistochemical analysis of 5hmC and 5caC distributions in the adult brain revealed that whereas the prominent 5hmC staining co-localizes with NeuN positive cells, NeuN-negative glial cells possess lower levels of genomic 5hmC (**Figure 1**)^20^.

We recently showed that Tet-dependent 5mC oxidation is operative during lineage specification of neural stem cells (NSCs)^ 20^. Despite being immunochemically undetectable in NSCs, 5fC and 5caC exhibit prominent immunostaining at early stages of NSCs differentiation towards neuronal and glial lineages. Both these marks transiently accumulate concurrently with appearance of the markers of early neuronal and glial differentiation^20^. To determine the distribution of 5caC in differentiating NSCs we performed co-staining of this mark with a glial marker GFAP on the fixed cultures of NSCs at 3 days of induction of glial differentiation. Unlike in NSCs or mature astrocytes (data not shown), we observed strong 5caC signal in relatively large proportion of the cells expressing GFAP in these cultures (**Figure 2**)^20^.


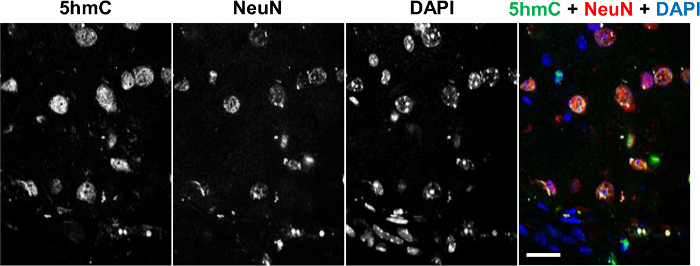
**Figure 1. Co-immunostaining of 5hmC (green) with NeuN (red) in the Adult Brain Tissue Counterstained with DAPI (Blue).** Individual channels and the merged view are shown. Scale bars are 25 µm. Please click here to view a larger version of this figure.


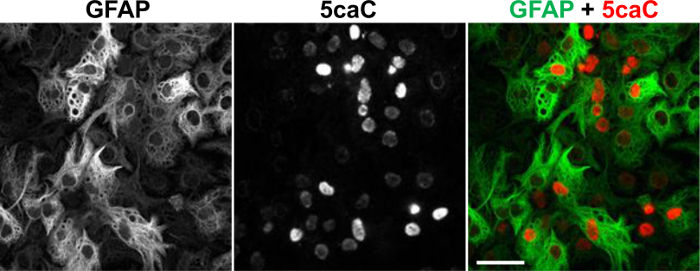
**Figure 2. Co-immunostaining of 5caC with GFAP in the Culture of NSC at Day 3 of Glial Differentiation.** Individual channels and the merged view are shown. Scale bars are 20 µm. Please click here to view a larger version of this figure.

## Discussion

Although the reported low abundance of 5mC oxidation derivatives, 5fC and 5caC in some tissues would present significant limitations for a standard immunochemistry protocol, the incorporation of peroxidase-conjugated secondary antibodies allowed the detection of these cytosine modifications in fixed tissues and cells (**Figure 2**). However, the optimal incubation time with the tyramide solution should be optimized experimentally for each individual batch of tyramide signal amplification kit where a linear relationship between signal intensity and duration of tyramide based signal amplification is observed, for details refer to Almeida *et al.* 2012^26^. In addition, the signal/background ratio can be significantly enhanced by carrying the washes in a Coplin jar to allow efficient removal of excess antibodies. Following incubation with tyramide solution, it is important to immediately stop the reaction by washing in PBT solution to decrease background staining. It is critical not to allow the sections to dry at any point during the procedure.

The efficiency of DNA depurination can be improved by carrying out the depurination reaction at 37 °C. While using 4 N HCl instead of 2 N enhances the staining of the modified forms of 5mC using 4 N HCl for DNA depurination would not permit co-staining with DAPI, as it interacts exclusively with double stranded DNA.

Although this technique can provide robust semi-quantitative assessment of the modified forms of cytosine bases where detectable, it cannot be used for evaluating the absolute levels of 5mC or its oxidation derivatives. Therefore, we recommend the use of other complementary but quantitative approaches^16, 19 -24^. Since the discovery of 5mC oxidation derivatives in mammalian genomes, several approaches have been developed to study their biological roles^16, 19-24^. Although these approaches can be valuable in determining the absolute levels of 5mC oxidation derivatives, they do not provide information concerning their spatial distribution^20, 26^. We have successfully used the method described here to map the spatial distribution and localization of 5mC oxidation derivatives in different cell types of the developing and adult brain^20^

By revealing their spatial distribution^20^, this technique can be crucial to understanding the biological implications and the fate of oxidized derivatives of 5mC in various biological contexts where these modified derivatives of 5mC can be detected including cellular differentiation, development and disease. In addition, the method we describe here can give semi-quantitative assessments of the oxidized derivatives of 5mC in different tissues by assessing the kinetics of peroxidase reaction (which is proportional to the staining intensity) at different incubation times with tyramide^26^.

## Disclosures

The authors declare no conflict of interest.
